# S100A8/A9 as a risk factor for breast cancer negatively regulated by DACH1

**DOI:** 10.1186/s40364-023-00548-8

**Published:** 2023-12-13

**Authors:** Xiaojun Zhang, Mengke Niu, Tianye Li, Yuze Wu, Jinnan Gao, Ming Yi, Kongming Wu

**Affiliations:** 1 General Surgery Department, Shanxi Bethune Hospital, Shanxi Academy of Medical Science, Tongji Shanxi HospitalThird Hospital of Shanxi Medical University, Taiyuan, China; 2 Department of Oncology, Tongji Hospital of Tongji Medical College, Huazhong University of Science and Technology, Wuhan, China; 3 Department of Gynecology, The Second Affiliated Hospital, College of Medicine, Zhejiang University, Hangzhou, China; 4 Department of Breast Surgery, The First Affiliated Hospital, College of Medicine, Zhejiang University, Hangzhou, China

**Keywords:** S100A8, S100A9, DACH1, Breast cancer, Prognosis, Biomarker

## Abstract

**Background:**

S100A8 and S100A9 are members of Ca^2+^-binding EF-hand superfamily, mainly expressed by macrophages and neutrophils. Limited by the poor stability of homodimers, they commonly exist as heterodimers. Beyond acting as antibacterial cytokines, S100A8/A9 is also associated with metabolic and autoimmune diseases such as obesity, diabetes, and rheumatoid arthritis. While the involvement of S100A8/A9 in breast cancer development has been documented, its prognostic significance and the precise regulatory mechanisms remain unclear.

**Methods:**

S100A8/A9 protein in breast cancer samples was evaluated by immunohistochemistry staining with tumor tissue microarrays. The serum S100A8 concentration in patients was measured by enzyme-linked immunosorbent assay (ELISA). The S100A8 secreted by breast cancer cells was detected by ELISA as well. Pooled analyses were conducted to explore the relationships between *S100A8/A9* mRNA level and clinicopathological features of breast cancer patients. Besides, the effects of S100A8/A9 and DACH1 on patient outcomes were analyzed by tissue assays. Finally, xenograft tumor assays were adopted to validate the effects of DACH1 on tumor growth and S100A8/A9 expression.

**Results:**

The level of S100A8/A9 was higher in breast cancer, relative to normal tissue. Increased S100A8/A9 was related to poor differentiation grade, loss of hormone receptors, and Her2 positive. Moreover, elevated S100A8/A9 predicted a worse prognosis for breast cancer patients. Meanwhile, serum S100A8 concentration was upregulated in Grade 3, basal-like, and Her2-overexpressed subtypes. Additionally, the results of public databases showed *S100A8/A9* mRNA level was negatively correlated to *DACH1*. Stable overexpressing DACH1 in breast cancer cells significantly decreased the generation of S100A8. The survival analysis demonstrated that patients with high S100A8/A9 and low DACH1 achieved the shortest overall survival. The xenograft models indicated that DACH1 expression significantly retarded tumor growth and downregulated S100A8/A9 protein abundance.

**Conclusion:**

S100A8/A9 is remarkedly increased in basal-like and Her2-overexpressed subtypes, predicting poor prognosis of breast cancer patients. Tumor suppressor DACH1 inhibits S100A8/A9 expression. The combination of S100A8/A9 and DACH1 predicted the overall survival of breast cancer patients more preciously.

**Supplementary Information:**

The online version contains supplementary material available at 10.1186/s40364-023-00548-8.

## Introduction

As the leading cause of cancer death among females all over the world, breast cancer draws intensive attention [1, 2]. Recently, the advancement of breast cancer molecular markers propels the innovation of prognosis evaluation and treatment strategy. Agents targeting human epidermal growth factor receptor 2 (Her2), hormone receptors, cyclin-dependent kinases, poly (ADP-ribose) polymerase, and immune checkpoints have completely changed the paradigm of breast cancer treatment [3–9]. Therefore, identifying key components contributing to the malignant biological properties is helpful to risk stratification and drug development for breast cancer patients [10–15].

S100A8 and S100A9 are members of Ca^2+^-binding EF-hand superfamily, mainly expressed by immune cells, especially macrophages and neutrophils [16]. Spontaneously, S100A8 and S100A9 form heterodimers or homodimers in vitro or in vivo, which could further generate heterotetramers in the presence of Ca^2+^ and/or Zn^2+^ [17]. Due to the identical expression pattern and poor stability of homodimers, S100A8 and S100A9 readily form heterodimers [17]. It is well known that S100A8/A9 plays an important role in defense against pathogen infection. Neutrophils or macrophages secret S100A8/A9 to magnify infection-induced inflammation by triggering advanced glycation end products (RAGE) and Toll-like receptor 4 (TLR4) signaling [18]. Subsequently, downstream signaling pathways such as NF-κB and MAPK are activated, and cytokines and chemokines are upregulated [19]. Moreover, S100A8/A9 has antibacterial properties by directly neutralizing Zn^2+^ and Mn^2+^, which are essential to bacteria growth [20]. Besides pathogen infection, S100A8/A9 is also associated with metabolic and autoimmune diseases such as obesity, diabetes, and rheumatoid arthritis [21]. Apart from a proinflammatory cytokine, S100A8/A9 acts as an intracellular Ca^2+^ sensor and regulates the activity of Ca^2+^-dependent biological behaviors, including cytoskeleton-membrane interaction and respiratory burst [22, 23]. Intact S100A8/A9 is the prerequisite for cell migration, exocytosis, phagocytosis, and reactive oxygen species (ROS) generation [17].

Notably, S100A8/A9 has the potential to promote cancer progression [24]. A low concentration of S100A8/A9 promotes cancer cell proliferation mainly by inducing phosphorylation of p38 and p44/42 MAPK [25]. Additionally, S100A8/A9 is positively correlated to breast cancer metastasis and chemoresistance [26, 27]. In addition to the activated intracellular signaling pathway, S100A8/A9-mediated accumulation of myeloid-derived suppressor cell (MDSC) impaires anti-tumor immune response and promoted tumor progression [27–29]. On the contrary, S100A8/A9 plays tumor suppressors in some types of cancers. In head and neck squamous cell carcinoma, S100A8/A9 is downregulated in mRNA and protein levels, and the loss of S100A8/A9 heterodimer is related to poor differentiation and the increased risk of metastasis. [30]. This S100A8/A9-attenuated tumorigenesis is mainly attributed to the arrested cell cycle and decreased matrix metalloproteinase 2 expression [30]. The role of S100A8/A9 in cancer progression needs further investigation. At present, although S100A8/A9 has been reported to participate in breast cancer development [31], rare studies are exploring the prognostic value of S100A8/A9 in breast cancer.

Besides, accumulating evidence demonstrates that DACH1 acts as a tumor suppressor in multiple types of cancers, including but not limited to breast cancer, non-small cell lung cancer [32, 33], gastric cancer [34], esophageal cancer [35], hepatocellular carcinoma [36, 37], renal carcinoma [38], and colorectal cancer [39]. DACH1 suppresses tumor progression by inhibiting cell proliferation, reversing oncogenic pathway-mediated malignant phenotypes of epithelial cells, hampering cell migration and invasion, reducing tumor stemness, inhibiting Forkhead signaling pathway, and enhancing the activity of p53 pathway [40–47]. The loss of DACH1 is frequently detected in various cancers and predicts the poor prognosis of cancer patients [48]. Notably, DACH1 could also inhibit malignant biological properties by regulating several cytokine-associated pathways, such as TGF-β, CXCL8, CXCL5, and CXCL1 [32, 34, 49–51].

This research aimed to delve deeper into the prognostic relevance of S100A8/A9 in the context of breast cancer. Our investigation involved an examination of the relationship between S100A8/A9 expression and various clinicopathological features of breast cancer. Furthermore, we devised a comprehensive prognostic model that combines S100A8/A9 with DACH1, a widely recognized tumor suppressor in breast cancer, to distinguish breast cancer patients with unfavorable prognoses.

## Materials and methods

### **Meta-analysis for the expression of S100A8 and S100A9 by gene expression omnibus (GEO) database**

We searched GEO database containing breast cancer patients in Array Express to assess the expression abundance of S100A8/A9 at the mRNA level. The datasets adopted met the following standards: (1) the dataset including breast cancer patients; (2) available mRNA abundance of S100A8/A9; (3) available clinical outcome or patients’ clinicopathological parameters; (4) adopting the latest and most complete datasets when datasets shared common patients [52]. After dataset selection, a total of 29 datasets were pooled in meta-analysis, as previously mentioned. Clinicopathological parameters included histological type, TNM stage, and estrogen receptor (ER)/progesterone receptor (PR)/Her2 status. The heterogeneity of meta-analysis was assessed by Cochrane-Q and inconsistency index (I^2^) statistics. The Stata software package (version 12.0) (Stata Corp LP, College Station, TX, USA) was applied in this meta-analysis [53].

### Bioinformatics analysis

The Cancer Genome Atlas (TCGA) breast cancer database was obtained from UCSC Xena browser (https://xenabrowser.net). We analyzed the correlations between mRNA abundance and clinicopathological parameters. Moreover, we plotted Kaplan-Meier survival curves the online tool Kaplan-Meier plotter (http://kmplot.com/analysis). Kaplan-Meier plotter could be utilized to evaluate the influence of 54,675 genes on the survival of 10,461 cancer samples. We analyzed overall survival (OS), relapse-free survival (RFS), and distant metastasis-free survival (DMFS) in breast cancer patients with different *S100A8/A9* levels. The Cancer Cell Line Encyclopedia (CCLE) dataset was also obtained from UCSC Xena browser, while the samples with low gene expression (RPKM < 1) were omitted in correlation analysis.

### Commercial tissue microarray (TMA)

Four commercial human breast cancer TMAs were purchased, including one BR2082a (Alenabio, Xi’an, China) and three HBre145Su01 (Outdo biotech, Shanghai, China). BR2082a consists of 32 metastatic breast cancer tissues obtained from lymph nodes, 120 breast cancer tissues obtained from primary organs, 8 fibroadenoma tissues, 16 hyperplasia breast tissues, 16 inflammation breast tissues, and 16 cancer adjacent normal tissues. HBre145Su01 includes 145 breast cancer tissue with 9-12.5 years of follow-up survival data.

### Immunohistochemical staining

Based on the two-step standard IHC protocol, immunohistochemical (IHC) staining was conducted to evaluate the abundances of S100A8, S100A9, and DACH1. Specifically, The process began with the removal of wax and rehydration of the slides. Slides were immersed in xylene and subsequently subjected to a series of graded alcohol solutions for rehydration. Then, slides underwent a series of steps: a triple rinse with TBS, antigen retrieval through heating, and another set of TBS rinses. Endogenous peroxidase activity was suppressed by applying 3% hydrogen peroxide, followed by a 10-minute incubation at room temperature and a final round of TBS rinses. To block non-specific binding, a 10% goat serum solution was applied for 30 min. The primary antibody was then introduced by adding 100 µl and allowing overnight incubation at 4 °C. The following day, slides were equilibrated to room temperature for 15 min, and another round of TBS rinses ensued. Subsequently, the secondary antibody was applied and allowed to incubate at room temperature for 35 min, followed by three TBS rinses. To visualize the target, 100 µl of freshly prepared DAB dye was added, and color development was observed under a microscope. The slides were rinsed under running water, counterstained with hematoxylin for 1 min, and rinsed again with running water. The slides were then dehydrated in a graded alcohol series, followed by drying, and finally sealed with xylene for transparency. Polyclonal anti-S100A8 antibody (15792-1-AP, Proteintech, 1:300), polyclonal anti-S100A9 antibody (26992-1-AP, Proteintech, 1:300), and polyclonal anti-DACH1 antibody (10914-1-AP, Proteintech, 1:200) were used as primary antibodies.

### Qualification analysis of IHC staining

Two experienced pathologists assessed IHC staining dependently by Fromowitz Standard. Few samples with poor staining quality were omitted in the qualification analysis. The staining intensity of the whole tissue was classified as 0 (no staining), 1 (weak staining, light-yellow), 2 (moderate staining, yellow-brown), and 3 (strong staining, brown). And the proportion of stained tumor cells was scored as 1 (0–25% stained cell), 2 (26–50% stained cell), 3 (51–75% stained cell), and 4 (76–100% stained cell). The multiplication of staining intensity and proportion was utilized to reflect the abundance of S100A8 and S100A9. IHC score ≥ median value was defined as high expression.

### Enzyme-linked immunosorbent assay (ELISA)

The blood samples were collected from patients diagnosed with breast cancer at Tongji Hospital from May 2018 to December 2018. About 85 patients who had not received surgery, radiotherapy, and chemotherapy were involved in the study. The concentration of S100A8 in serum was evaluated with Human S100A8 DuoSet ELISA kit (DY4570-05, R&D system) following the manufacturer’s recommendations. Briefly, S100A8 standards with known concentration, control samples, and patient serum samples were diluted and pipetted into these wells. Then, the second biotinylated monoclonal antibody and Streptavidin-Peroxidase were added. After washing unbound substances in the microplate, the substrate solution (TMB) was added. Following the reaction stop, the values of optical densities (OD) were measured at 450 nm through Microplate Reader (BioRad). The intensity of OD proportionated to the abundance of S100A8 in serum.

### Cell culture and transfection

Human breast cancer cells (CAMA-1 and MDA-MB-231) were sourced from Laboratory of Oncology Department, Tongji Hospital and maintained in RPMI-1640 medium containing 10% fetal bovine serum. Cells were cultured in humidified atmosphere with 5% CO_2_ at 37 °C. As the previous description, plasmids encoding DACH1 were subcloned into lentivirus expression vectors [50]. Stable sublines expressing DACH1 and an empty vector control were generated through transient co-transfection of the DACH1-expressing vector with packaging plasmids in HEK-293T cells. After 48- and 72- hours post-transfection, the culture medium was harvested and subsequently filtered using a 0.45 μm filter for the infection of breast cancer cells, in the presence of 8 mg/ml polybrene. The pool of transduced cancer cells was further selected by a two-week Puromycin treatment at a concentration of 2 ug/ml. Cells with stable DACH1 expression were identified by protein level as previously described [43]. Then, supernant was collected for ELISA assay.

### Western blotting (WB)

Breast cancer cells were collected and lysed with Total Protein Extraction Kit (DE101-01, Transgen). Protein (25 µg per sample) was separated by SDS-PAGE assay. In brief, samples was mixed with Loading Buffer, followed by a 10-minute heating at 95 °C and subsequent cooling for later use. Once the gel concentrate had solidified and lanes were gently rinsed with the electrophoretic buffer before adding the samples. Electrophoresis commenced at a constant 90 V, transitioning to 120 V when Bromophenol Blue reached the separation gel’s bottom. Following this, a PVDF membrane, similarly sized to the gel, was soaked in methanol. Two sheets of Bio-Rad semi-dry transfer thick filter paper were soaked in the transfer solution. The transfer gel sandwich, assembled with filter paper, PVDF membrane, gel, and more filter paper, underwent careful bubble removal, and semi-dry transduction began at 15 V. The PVDF membrane was placed in Ponceau staining solution, leading to the emergence of visible protein bands upon successful membrane transfer. Subsequent steps involved washing, incubating with primary antibodies, washing again, incubating with secondary antibodies, and finishing with development and imaging in a Bio-Rad gel imaging system. The primary antibodies included anti-DACH1 antibody (1:200, 10914-1-AP, Proteintech) and anti-GAPDH (1:1000, 5174, CST). Anti-rabbit-IgG-HRP (1:2000, 7074, CST) was used as the secondary antibody, and the signal was detected with Chemidoc System (Bio-Rad) [54].

### Luciferase reporter gene assay

Plasmids encoding DACH1, DACH1 DS-domain deleted (ΔDS) (including an N-terminal Flag peptide), and c-terminal (C-ter) have been previously documented [50]. The primers employed for synthesizing the S100A8 promoter sequence are provided below: Forward primer, 5’- ggtaccgagctcttacgcgtctttgtaacaacagaaacacaccaca-3’; Reverse primer, 5’- tacttagatcgcagatctcgagGACAGCTGACAAGAGACATgc-3’. Correspondingly, the primers for S100A9 promoter sequence synthesis are: Forward primer, 5’-ggtaccgagctcttacgcgt ccgagagggtcaggcccccataggtcctcag-3’; Reverse primer, 5’-tacttagatcgcagatctcgag GCGACATTTTGCAAGTCATCG-3’. The sequences of S100A8/A9 promoters were inserted into pGL3-basic vectors using Mlu I and Xho I restriction sites. 293T cells were seeded at a density of 1 × 10^5^ cells in a 24-well plate one day prior to transfection. The DACH1 expressing plasmid or its mutants were combined with the Luciferase reporter plasmid for transient transfection. Following a 36-hour incubation, cells were harvested, and luminescence was measured using the Bio-Lite™ Luciferase Assay System (DD1201, Vazyme).

### Xenograft model

BALB/c Nude mice were inoculated with 3 × 10^6^ MDA-MB-231-vector or MDA-MB-231-DACH1 cells in the right mammary fat pad. Tumor size was measured every five days with a digital caliper. The volume was calculated based on the following formula: 0.5×length×width^2^. Mice were euthanized on day 35 after implantation.

### Statistical analysis

To compare two groups, we utilized Student’s t-test with or without Welch’s correction and the Mann-Whitney test. Differences among multiple groups were analyzed by one-way ANOVA. Group were analyzed by Student’s t test and one-way ANOVA. *P* value below 0.05 was regarded as statistical significance. Correlations between IHC scores and clinic-pathological features were analyzed by Pearson chi-square test. The survival curves were compared with Log-rank test. All statistical analyses were conducted with R software (4.1.2) and GraphPad Prism (9.0).

## Results

### The abundances of S100A8 and S100A9 were increased in Breast cancer

**Fig. 1:**
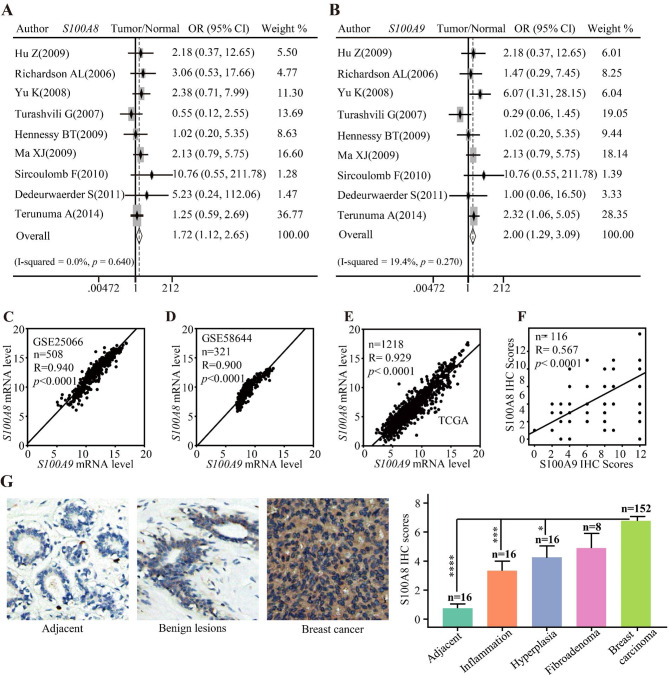
The expression levels of S100A8/A9 in normal breast, benign disease, and breast cancer tissues. (**A**–**B**) Pooled analysis of GEO datasets showing *S100A8/A9* mRNA level in normal tissues and breast cancers. (**C**–**E**) Scatter plots showing the correlations between *S100A8* and *S100A9* mRNA levels using three datasets. (**F**) Scatter plots showing the correlation between S100A8 and S100A9 protein levels using IHC staining scores. (**G**) The representative IHC staining images showing S100A8 protein expression in normal breast, benign disease, and breast cancer tissues. IHC: immunohistochemical

Based on 29 GEO datasets (Additional file1: Table S1), a series of meta-analyses were conducted to investigate the differences in S100A8 and S100A9 expressions between normal tissue and breast cancer. The results showed that *S100A8* and *S100A9* mRNA increased significantly in tumor compared with normal breast tissue (Fig. 1A–B). Moreover, we found *S100A8* mRNA has an identical expression pattern to *S100A9* mRNA as previously reported. Here, two gene expression datasets (GSE25066 and GSE58644) and TCGA database were used to analyze the correlation. The result showed that *S100A8* positively correlated with *S100A9* in mRNA level (*P* < 0.0001) (Fig. 1C–E). Besides, the IHC staining of S100A8 and S100A9 using two TMAs (both were HBre145Su01) showed that S100A8 significantly correlated with S100A9 at the protein level as well (*P* < 0.0001) (Fig. 1F). Therefore, we used S100A8 as the surrogate to explore the expression of S100A8/A9 in breast cancer. The IHC staining using one TMA (BR2082a) was conducted to interrogate S100A8 expression in normal and tumor tissues. We found that S100A8 abundance is significantly higher in breast cancer tissue than normal breast tissue (*P* < 0.0001), breast inflammation (*P* = 0.0006), and hyperplasia breast tissue (*P* = 0.0130) (Fig. 1G). Also, we observed an upward trend in cancer tissue compared to fibroadenoma, although this trend did not reach statistical significance (*P* = 0.1742).

### The levels of S100A8 and S100A9 correlated with molecular subtypes of Breast cancer

**Fig. 2:**
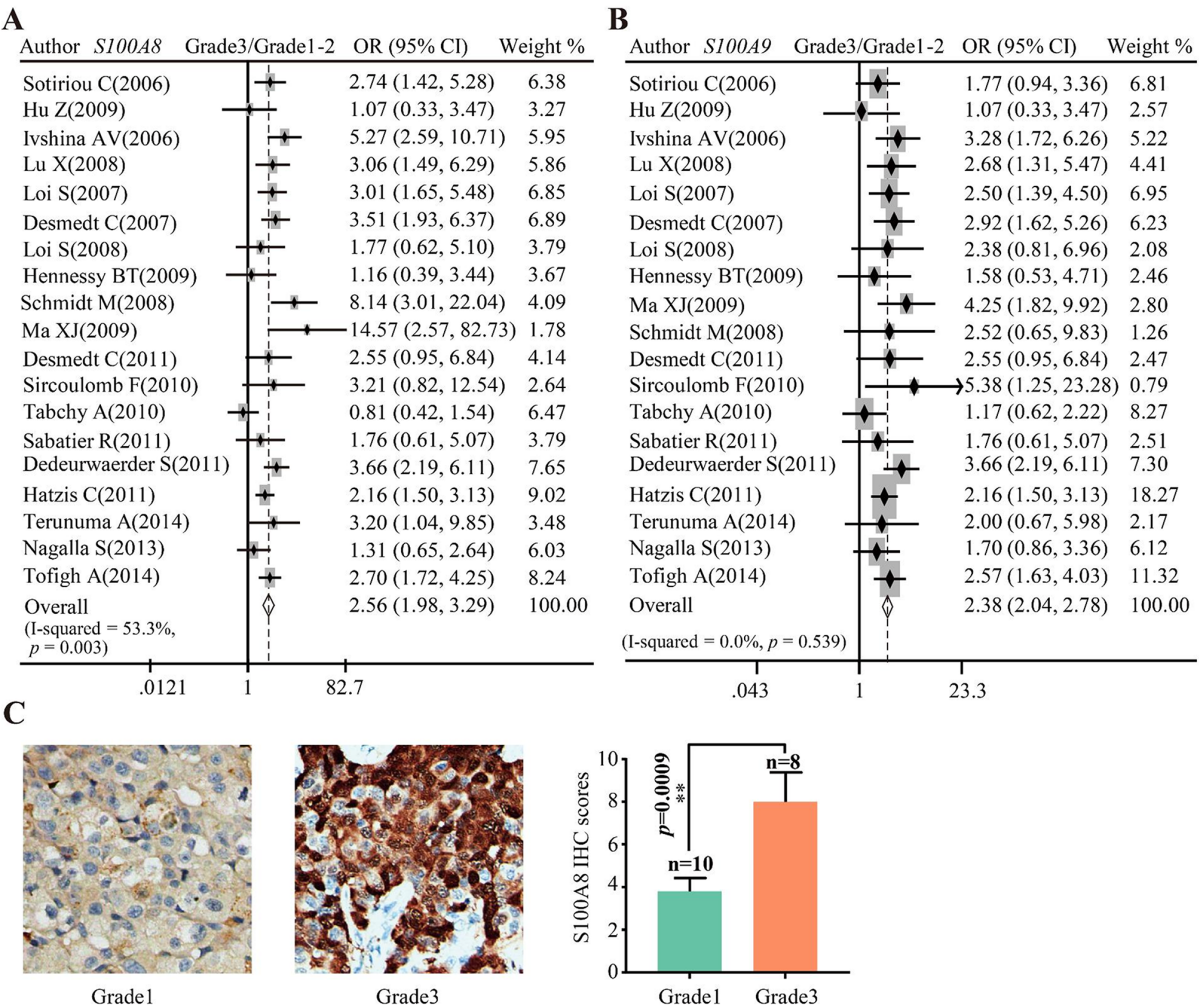
The relationships between S100A8/A9 expression and tumor differentiation grade. (**A**–**B**) Pooled analysis of GEO datasets showing the relationship between *S100A8*/*A9* mRNA level and differentiation grade. (**C**) The representative IHC staining images showing S100A8 protein expression in grade1 and grade3 breast cancers. IHC: immunohistochemical

The results of pooled analysis showed that both *S100A8* and *S100A9* mRNA abundances were dramatically upregulated in grade 3 breast cancer tissues in comparison with grade 1–2 cancer tissues (Fig. 2A–B). Consistent with the trend of analysis in mRNA level, the IHC staining of S100A8 using two TMAs (BR2082a and HBre145Su01) showed that S100A8 expression was significantly upregulated in poorly differentiated tumor tissues (grade3/grade1: *P* = 0.0009, Fig. 2C).

**Fig. 3:**
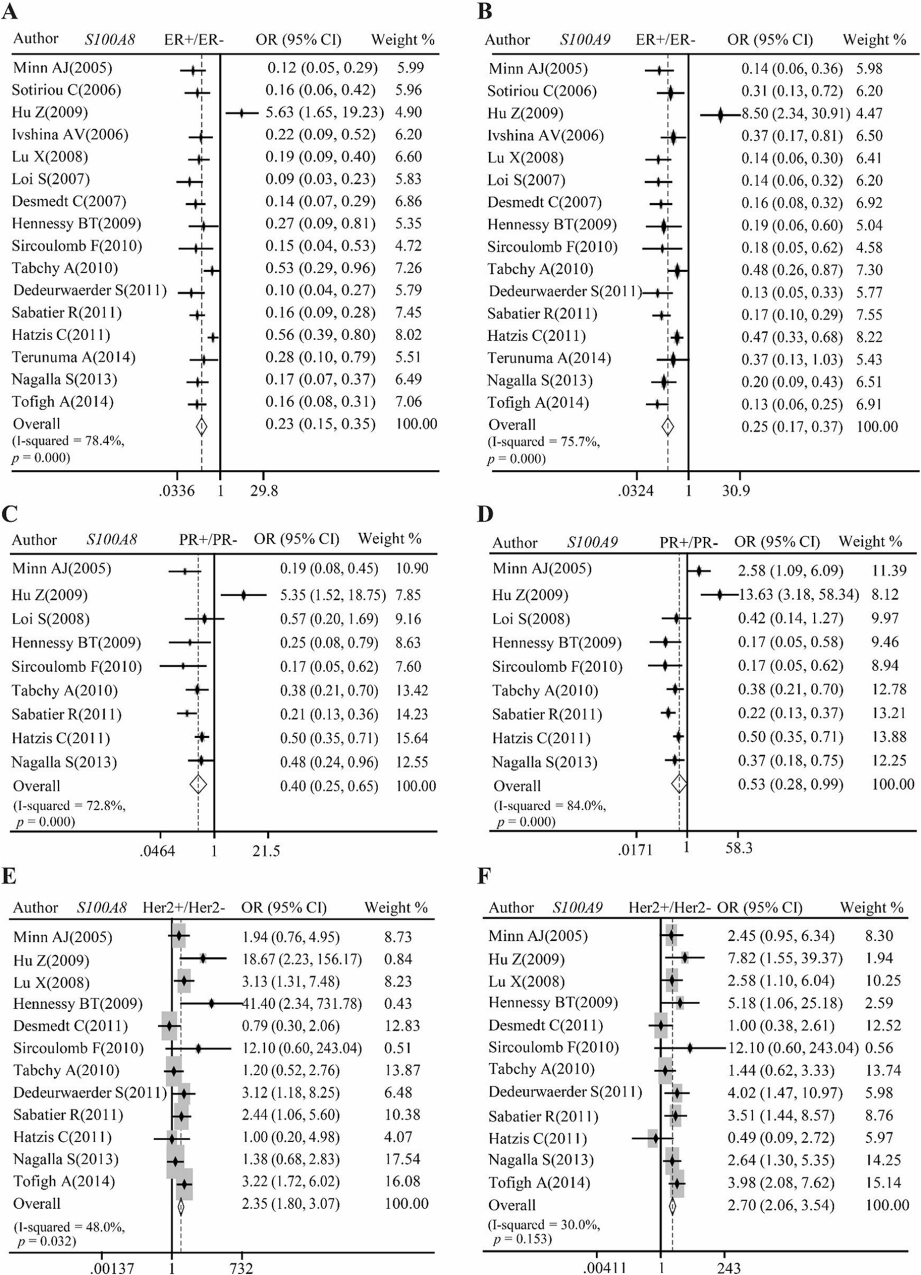
**Pooled analysis measuring relationships between** ***S100A8/A9*** **mRNA and molecular biomarkers of breast cancers. (A-B)** Pooled analysis of GEO datasets showing relationships between *S100A8/A9* mRNA level and estrogen receptor status. **(C-D)** Pooled analysis of GEO datasets showing relationships between *S100A8/A9* mRNA level and progesterone receptor status. **(E-F)** Pooled analysis of GEO datasets showing relationships between *S100A8/A9* mRNA level and Her2 status

**Fig. 4:**
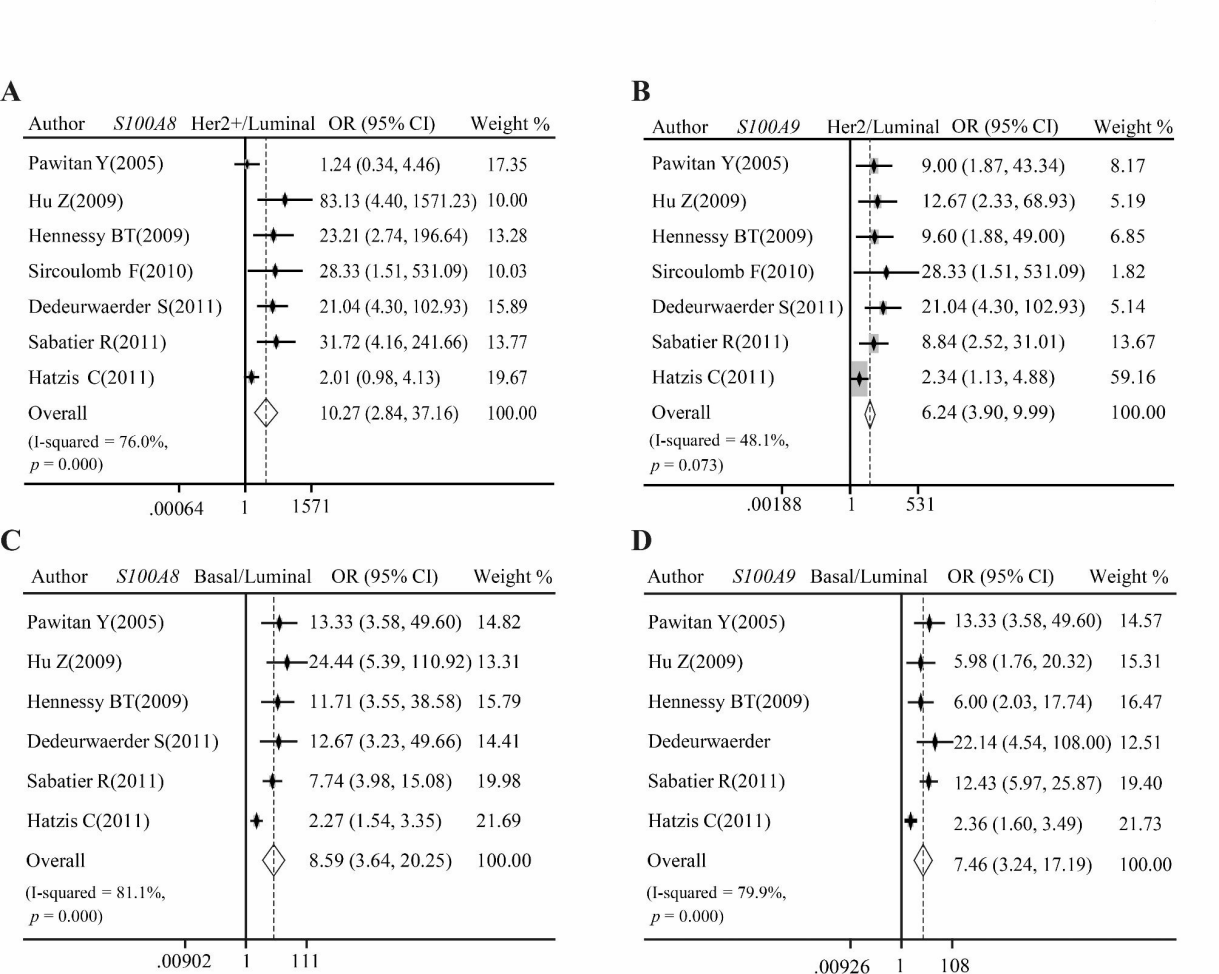
Pooled analysis measuring relationships between *S100A8/A9* mRNA and subtypes of breast cancers. (**A**–**B**) Pooled analysis of GEO datasets showing *S100A8/A9* mRNA level in Her2-overexpressed and Luminal breast cancers. (**C**–**D**) Pooled analysis of GEO datasets showing *S100A8/A9* mRNA level in Basal-like and Luminal breast cancers

We further explored *S100A8* and *S100A9* mRNA levels in patients with different ER, PR, and Her2 statuses. Notably, the meta-analysis showed that the significant downregulation of *S100A8* and *S100A9* mRNA in ER+ (Fig. 3A–B) and PR+ (Fig. 3C–D), but upregulation in Her2+ (Fig. 3E–F) group. In addition, analysis based on TCGA breast cancer RNA-seq datasets showed the same trend that *S100A8* and *S100A9* mRNA was upregulated in ER-, PR-, and Her2 + patients (*P* < 0.0001, Additional file 2: Figure S1A–B). We subsequently evaluated *S100A8* and *S100A9* mRNA expression in different molecular subtypes which were primarily determined by biomarkers such as ER, PR, and Her2. The pooled analysis indicated *S100A8* and *S100A9* mRNA were increased in Her2-overexpressed subtype (Fig. 4A–B) and basal subtype (Fig. 4C–D), relative to luminal subtype. The data from the TCGA breast cancer datasets showed similar trends (*P* < 0.0001, Additional file 2: Figure S1C–D).

**Fig. 5:**
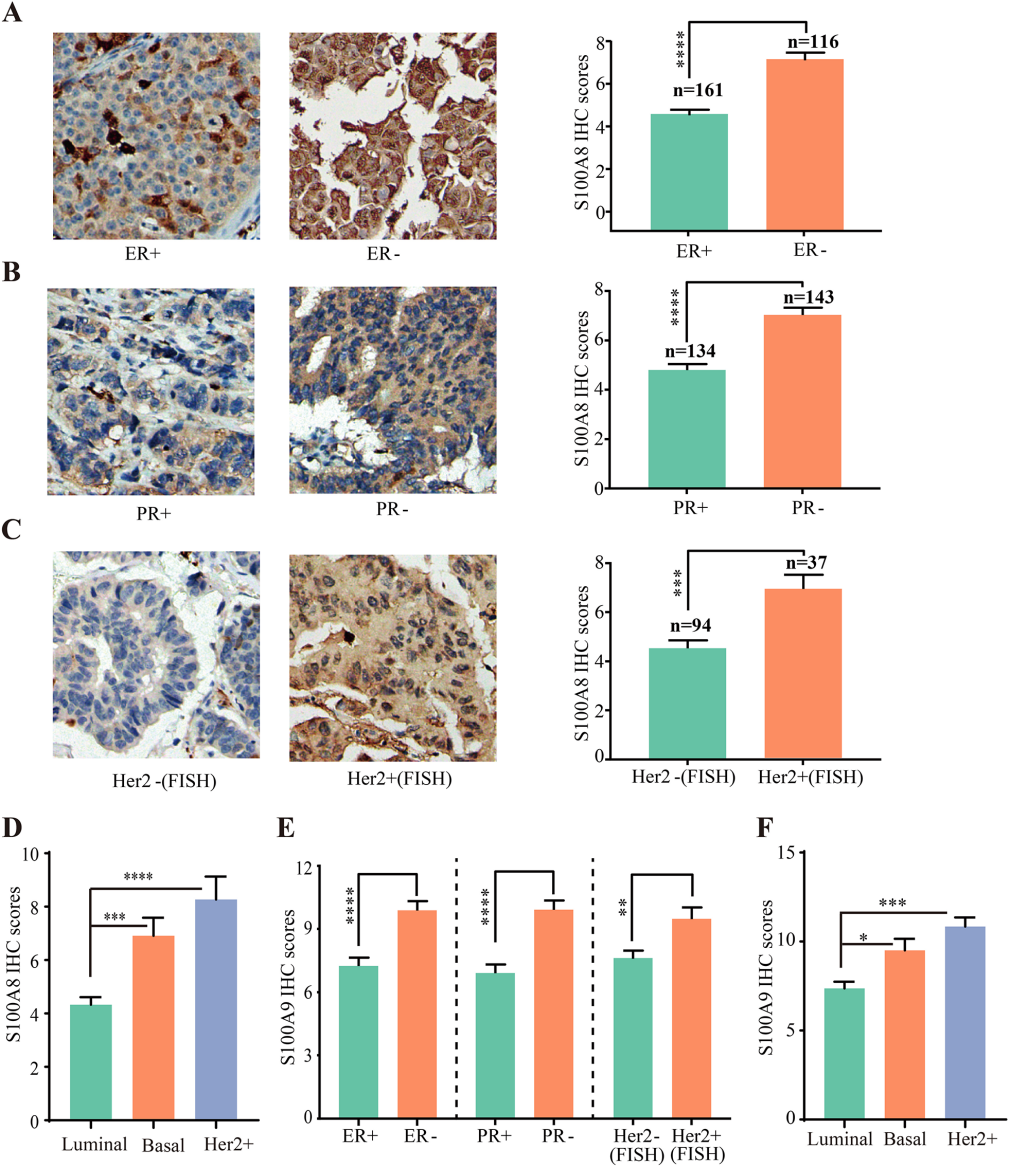
IHC staining showing the relationship between S100A8/A9 protein level and subtypes of breast cancers. (**A**) The representative IHC staining images showing S100A8 protein expression in ER + and ER- breast cancers. (**B**) The representative IHC staining images showing S100A8 protein expression in PR + and PR- breast cancers. (**C**) The representative IHC staining images showing S100A8 protein expression in Her2 + and Her2- breast cancers. (**D**) Histogram showing S100A8 protein expression in different subtypes of breast cancers. (**E**) Histogram showing S100A9 protein level in ER+, ER-, PR+, PR-, Her2+, and Her2- breast cancers. (**F**) Histogram showing S100A9 protein level in different subtypes of breast cancers. ER: estrogen receptor; PR: progesterone receptor; IHC: immunohistochemical

Then, the S100A8 protein level in different subtypes was evaluated. The abundance of S100A8 was remarkably correlated with the statuses of hormone receptors and Her2. S100A8 was significantly upregulated in ER- (*P* < 0.0001), PR- (*P* < 0.0001), and Her2+ (*P* = 0.0003) tumors (Fig. 5A–C). Further analysis revealed that S100A8 was remarkably upregulated in basal-like (*P* = 0.0002) and Her2 overexpressed (*P* < 0.0001) subtype tumors in comparison with luminal subtype tumors (Fig. 5D). We further analyzed overall clinicopathological data, and no other factors had significant statistical correlations with S100A8 (Table 1).

**Table 1:** Correlation between S100A8 expression and clinicopathological features of breast cancer patients in two TMAs (pooled data of BR2082a and HBreD145Su01)

Variables	n	S100A8 (High)	S100A8 (Low)	*P* value
Age				0.475^a^
≥median	153	75	78	
<median	141	75	66	
Grade				0.039^b^
3	8	5	3	
2 + 2 ~ 3	193	107	86	
1 + 1 ~ 2	36	12	24	
Tumor size				0.188^a^
T3-T4	31	19	12	
Tis-T2	230	112	118	
Lymph node				0.108^a^
N+	124	56	68	
N-	136	75	61	
Stage				0.729^a^
III	69	36	33	
I–II	191	95	96	
ER				< 0.0001^a^
ER+	161	64	97	
ER-	116	79	37	
PR				< 0.0001^a^
PR+	134	51	83	
PR-	143	91	52	
Her2				0.002^a^
Her2+	37	31	6	
Her2-	94	51	43	

^a^Pearson chi-square test, ^b^Fisher’s exact test

Additionally, IHC staining assays were conducted with another TMA (HBre145Su01) to evaluate the expression of S100A9 in different molecular subtype breast cancers. Similar to S100A8, S100A9 expression was significantly upregulated in ER- (*P* < 0.0001), PR- (*P* < 0.0001), Her2+ (*P* = 0.00097) breast cancer (Fig. 5E) (Additional file 1: Table S2). Correspondingly, S100A9 expression was higher in basal-like (*P* = 0.016) and Her2-overexpressed (*P* = 0.0006) samples, relative to luminal tumors (Fig. 5F).

### High S100A8 and S100A9 levels correlated to the poor prognosis of Breast cancer patient

**Fig. 6:**
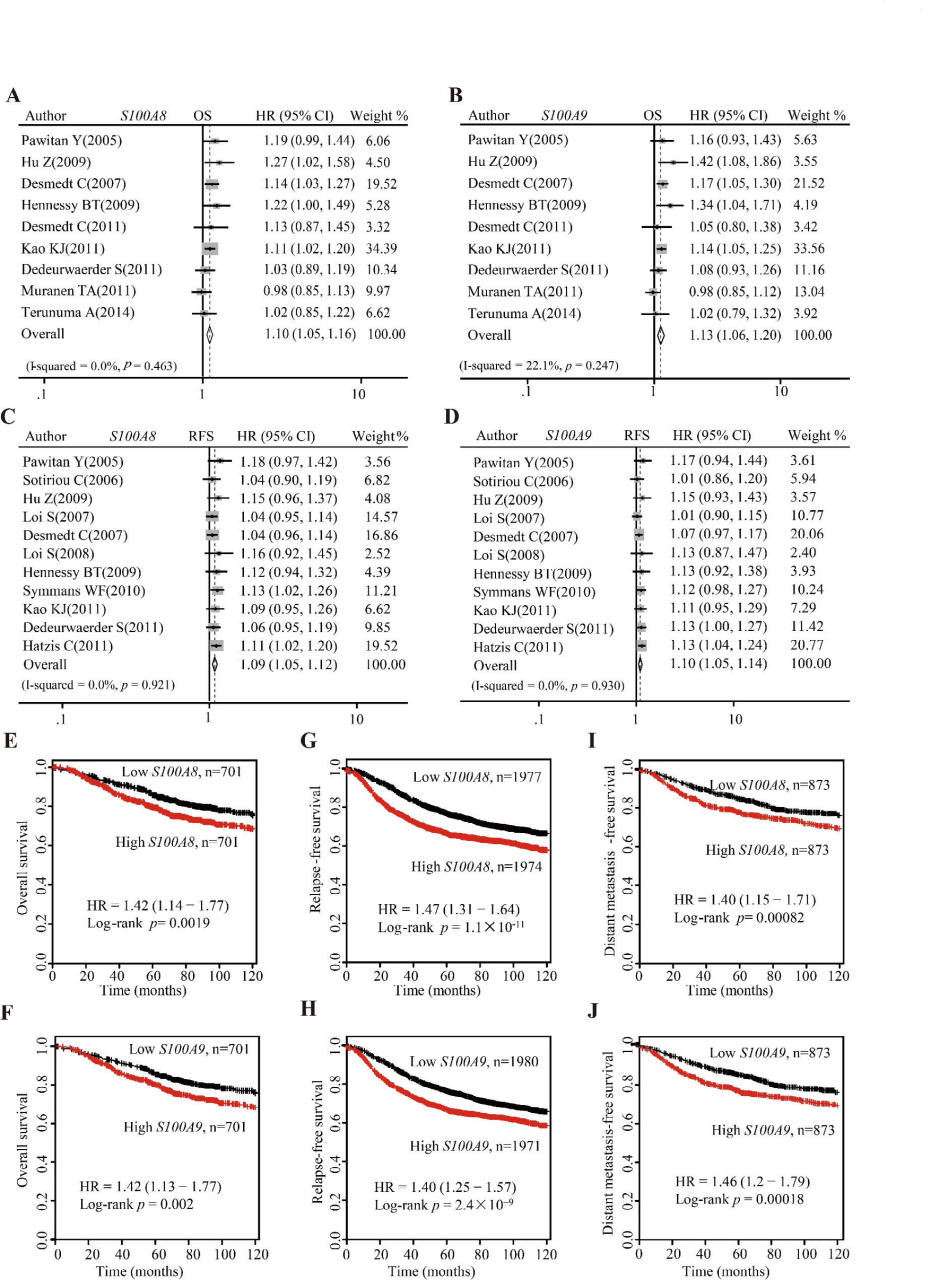
The predictive values of *S100A8/A9* mRNA levels for the outcomes of breast cancer patients. (**A**–**B**) Pooled analysis using GEO datasets showing the relationships between *S100A8/A9* mRNA levels and OS. (**C**–**D**) Pooled analysis using GEO datasets showing the relationships between *S100A8/A9* mRNA levels and RFS. (**E**–**F**) Survival curves from Kaplan-Meier plotter showing the relationships between *S100A8/A9* mRNA levels and OS. (**G**–**H**) Survival curves from Kaplan-Meier plotter showing the relationships between *S100A8/A9* mRNA level and RFS. (**I**–**J**) Survival curves from Kaplan-Meier plotter showing the relationships between *S100A8/A9* mRNA levels and DMFS. OS: overall survival; RFS: relapse-free survival; DMFS: distant metastasis-free survival

Based on the public GEO database and online analysis tool Kaplan-Meier plotter, we interrogated the relationship between *S100A8/A9* mRNA level and outcomes of breast cancer patients including OS, PFS, DMFS, and DFS. Meta-analysis revealed that increased *S100A8* and *S100A9* mRNA expression were related to poor OS (Fig. 6A–B), RFS (Fig. 6C–D), DMFS (Additional file 3: Figure S2A–B), and DFS (Additional file 3: Figure S2–D). Moreover, the result of Kaplan-Meier survival analysis indicated that high *S100A8* and *S100A9* mRNA levels correlated to poor OS (Fig. 6E–F), RFS (Fig. 6G–H), and DMFS (Fig. 6I–J).

**Fig. 7:**
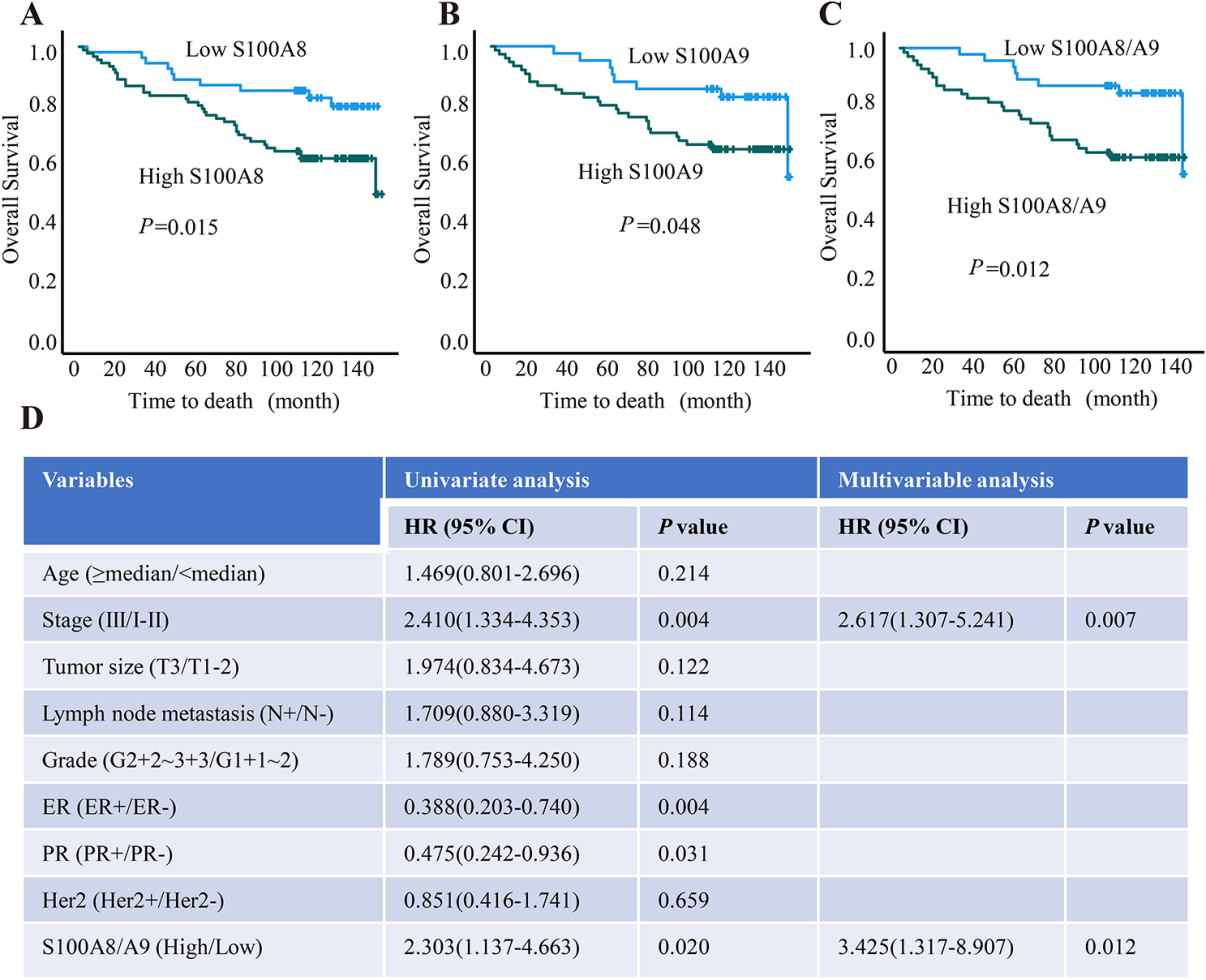
The predictive values of S100A8/A9 protein level for the outcomes of breast cancer patients. (**A**–**C**) The impact of S100A8 and S100A9 proteins, as well as their average values, on overall survival of breast cancer patients. (**D**) Cox univariate regression and Cox multivariable regression analysis between cumulative overall survival rate and clinicopathological variables of breast cancer patients. OS: overall survival

Then, we carried out Kaplan-Meier survival analysis to explore the relationship between S100A8 protein level and OS during a follow-up period of up to 12 years. Based on survival data of TMA HBre145Su01, we found that the high S100A8 expression group (IHC score ≥ median value) had poor OS compared with the low S100A8 expression group (IHC score < median value) (Fig. 7A). Similarly, the result of Kaplan-Meier survival analysis showed that high S100A9 and S100A8/A9 mean value were unfavorable factors for the OS of breast cancer patients (Fig. 7B–C). Moreover, we conducted a Cox univariate regression to investigate the influence of clinicopathological factors on cumulative OS. TNM stage, ER status, PR status, and S100A8/A9 expression (the average value of S100A8 and S100A9 expression) were found significantly related to OS. We further performed a Cox multivariable regression analysis which included candidate variables with *P* < 0.2 in Cox univariate regression. The multivariable analysis revealed that S100A8/A9 expression is an independent prognostic factor of OS for breast cancer patients (HR = 3.425, 95%CI 1.317–8.907) (*P* = 0.012, Fig. 7D).

### S100A8 concentration in the serum correlated to the clinicopathological features of Breast cancer patients

**Fig. 8:**
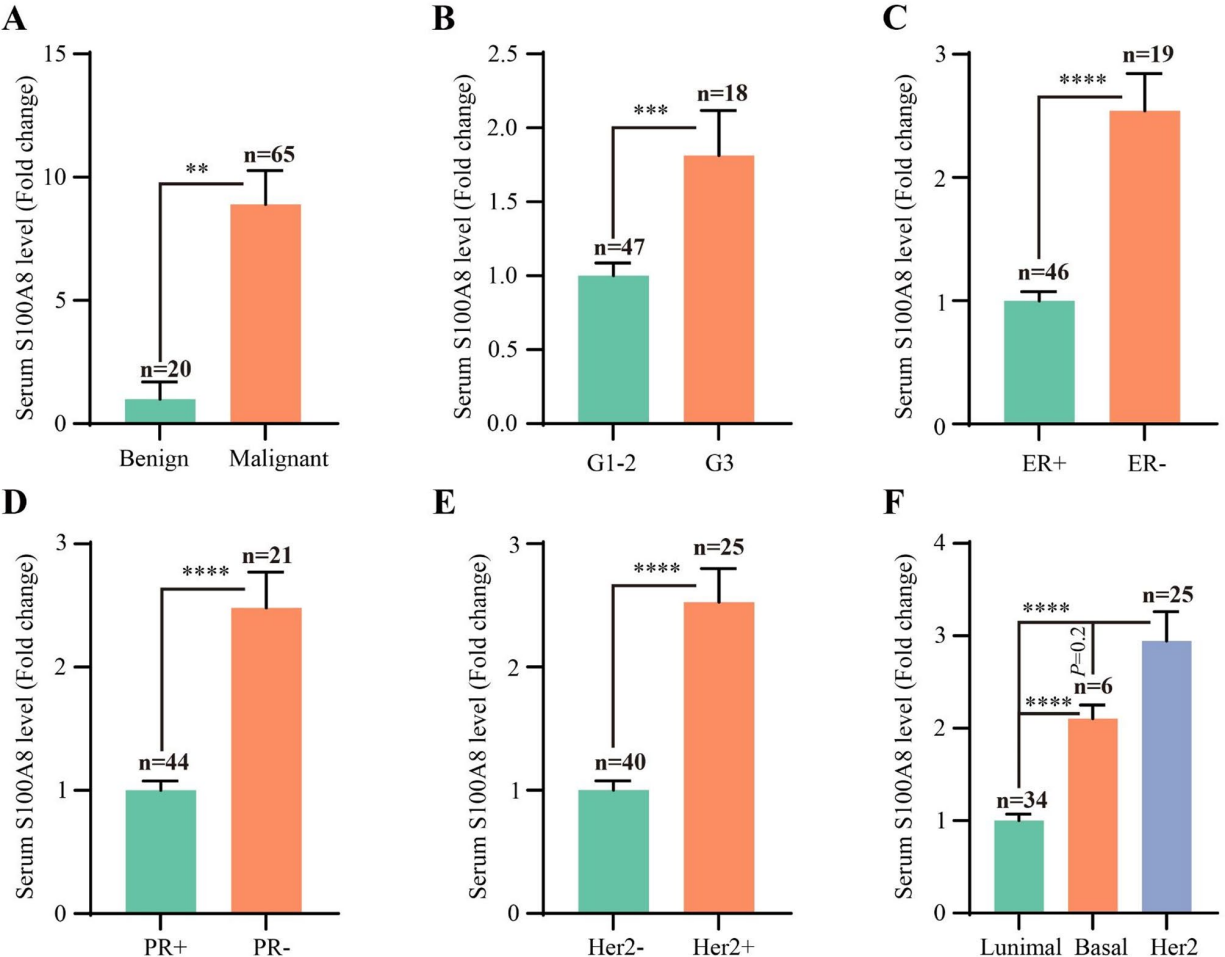
The relationship between serum S100A8 level and clinicopathological features of breast cancers. (**A**) Histogram showing serum S100A8 level of patients with benign breast nodules and breast cancers. (**B**) Histogram showing the relationships between serum S100A8 level and tumor differentiation grade. (**C**–**E**) Histogram showing the relationships between serum S100A8 level and ER, PR, and Her2 status. (**F**) Histogram showing the relationships between serum S100A8 level and subtypes of breast cancers. ER: estrogen receptor; PR: progesterone receptor

We measured S100A8 concentration in the serum of 85 patients. The tested samples were from 20 patients with benign solid breast nodules and 65 patients with breast cancers (Additional file 1: Table S3). The results showed that serum S100A8 concentration was higher in breast cancer patients, relative to patients with benign breast nodules (*P* = 0.002, Fig. 8A). Moreover, increased serum S100A8 level was associated with poor differentiation (*P* = 0.0009, Fig. 8B), ER- (*P* < 0.0001, Fig. 8C), PR- (*P* < 0.0001, Fig. 8D), Her2+ (*P* < 0.0001, Fig. 8E), and basal/Her2-overexpressed subtypes (*P* < 0.0001, Fig. 8F). Collectively, high S100A8 level in serum was closely associated with clinicopathological features of breast cancer patients, predicting poor prognosis.

### DACH1 reduced the expression of S100A8 and S100A9

**Fig. 9:**
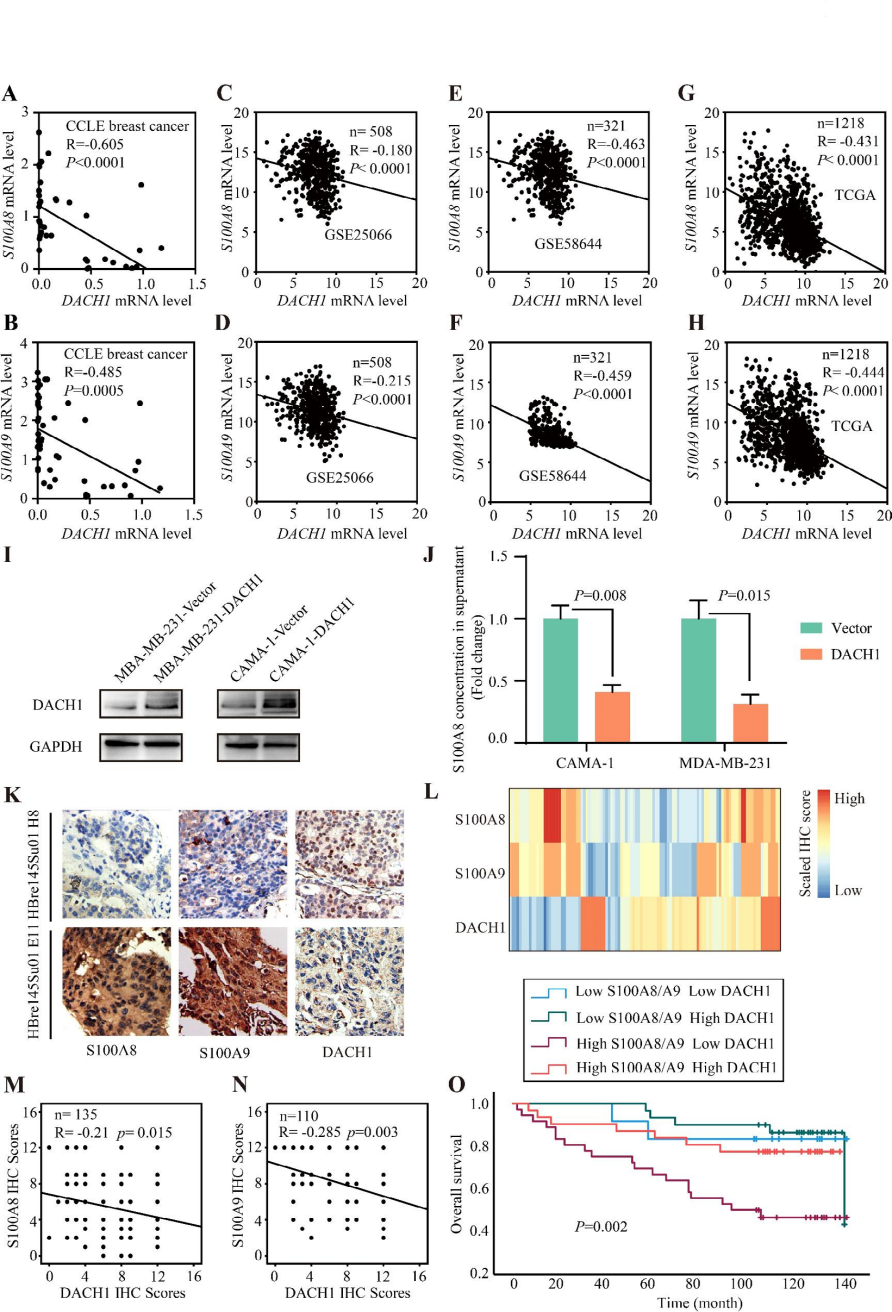
The regulation effect of DACH1 on S100A8/A9 expression. (**A**–**H**) Scatter plots showing the correlations between *S100A8*/*A9* and *DACH1* mRNA levels using four datasets. (**I**) Western blotting assays showing the DACH1 protein level in CAMA-1 and MDA-MB-231 cells. (**J**) ELISA demonstrated that DACH1 inhibited S100A8 expression in CAMA-1 and MDA-MB-231 cells. (**K**) The representative IHC staining images showing the expression levels of S100A8, S100A9, and DACH1 in the same sample. (**L**) Heatmap showing the IHC scores of S100A8, S100A9, and DACH1 in IHC staining assays. (**M**–**N**) Scatter plots showing the correlations between S100A8/A9 and DACH1 protein levels using IHC scores. (**O**) The blend Kaplan-Meier curves of S100A8/A9 and DACH1 with OS of breast cancer patients of tissue microarrays. OS: overall survival; IHC: immunohistochemical

DACH1 is a well-established suppressor of breast cancer [55], and the loss of DACH1 is associated with malignant biological properties of breast cancer [42]. Here, we analyzed the correlation among *S100A8*, *S100A9*, and *DACH1* mRNA expression in breast cancer. Three gene expression datasets (CCLE, GSE25066, and GSE58644) and TCGA database were used to analyze the correlation. The results showed that both *S100A8* and *S100A9* mRNA levels negatively were correlated with *DACH1* (Fig. 9A–H). To explore the effects of DACH1 on S100A8/A9 expression, two breast cancer cell lines with medium-low endogenous DACH1 expression were transfected with control or DACH1 plasmids [56]. The supernatants of CAMA-1-vector, CAMA-1-DACH1, MDA-MB-231-vector, and MDA-MB-231-DACH1 were collected to measure the concentration of S100A8 by ELISA. The results showed that DACH1 decreased the expression of S100A8 in CAMA-1-DACH1 and MDA-MB-231-DACH1, relative to controls (Fig. 9I and J). Additionally, the IHC staining also demonstrated that S100A8 and S100A9 were negatively correlated with DACH1 expression in breast cancer samples (Fig. 9K–N).

**Fig. 10:**
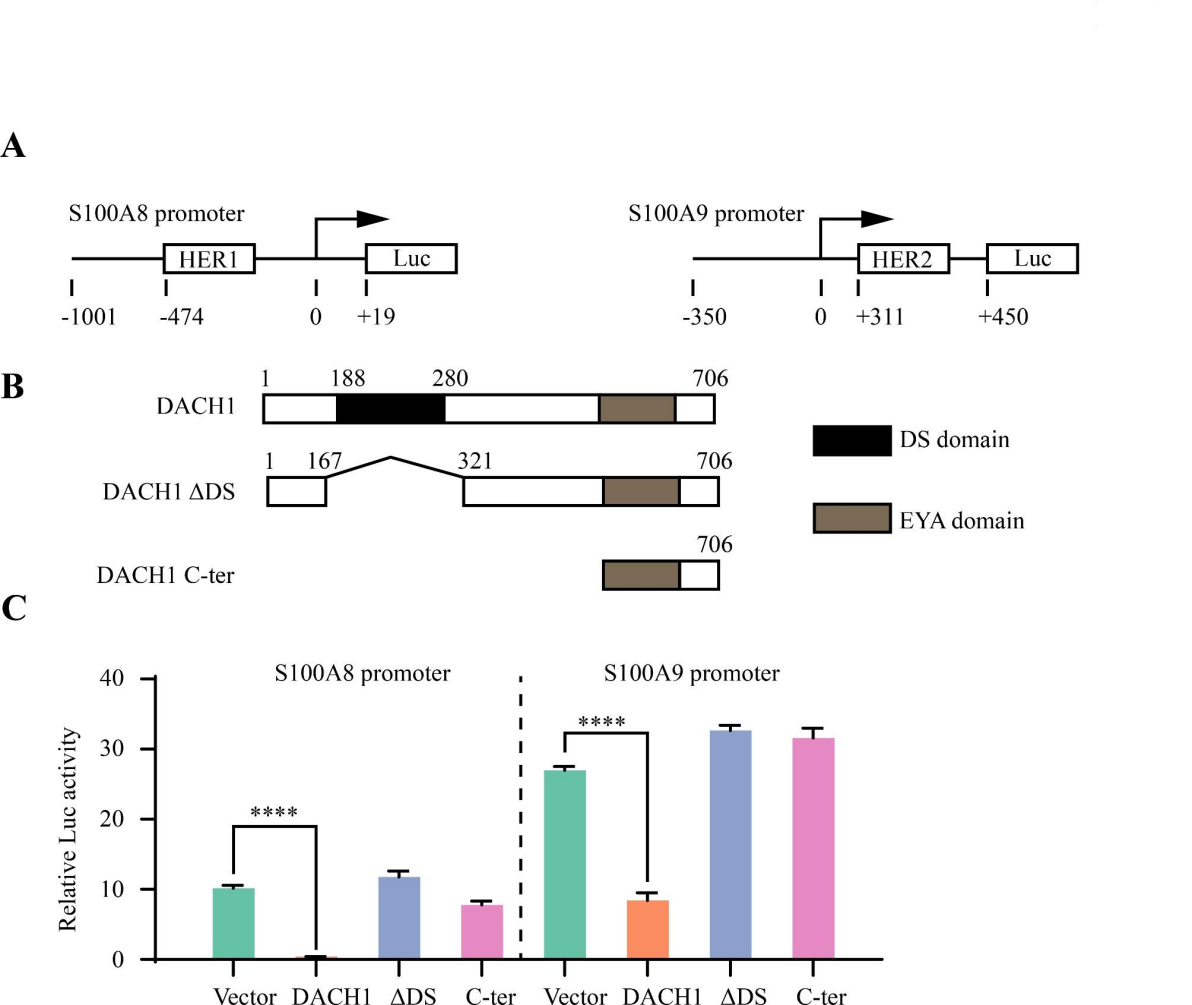
DACH1 suppressed the expression of S100A8/A9 via its DS domain. (**A**) Diagram illustrating the S100A8/A9 promoter plasmids employed in luciferase reporter gene assays. (**B**) Schematic representation of the DACH1 expressing plasmid and its mutants. (**C**) Relative luciferase activity of the S100A8/A9 promoter in 293T cells expressing DACH1 or its mutants. Luminescence values were normalized against the pGL3-basic vector

To further explore the clinical significance of S100A8/A9 and DACH1 levels in breast cancer, we performed a combined analysis using the IHC staining scores of S100A8, S100A9, and DACH1. Due to the identical expression pattern of the two proteins, the average scores of S100A8 and S100A9 were adopted as the expression level of S100A8/A9. The results showed that patients with high S100A8/A9 and low DACH1 achieved the shortest median OS (*P* = 0.002, Fig. 9O). Collectively, the combination of S100A8/A9 and DACH1 expression predicted patient prognosis more precisely. Next, in order to elucidate the mechanisms underlying DACH1-mediated inhibition of S100A8/A9 expression, various mutant DACH1 expression plasmids were investigated for their effects on the S100A8/A9 promoters (Fig. 10A–B). Through luciferase reporter gene assays, we assessed the activities of S100A8/A9 promoters in the presence of different DACH1 expression plasmids. Our findings indicate that DACH1 predominantly suppresses the S100A8/A9 promoter through its DS domain (Fig. 10C).

### DACH1 suppressed Breast cancer growth and negatively correlated with S100A8/A9 expression

**Fig. 11:**
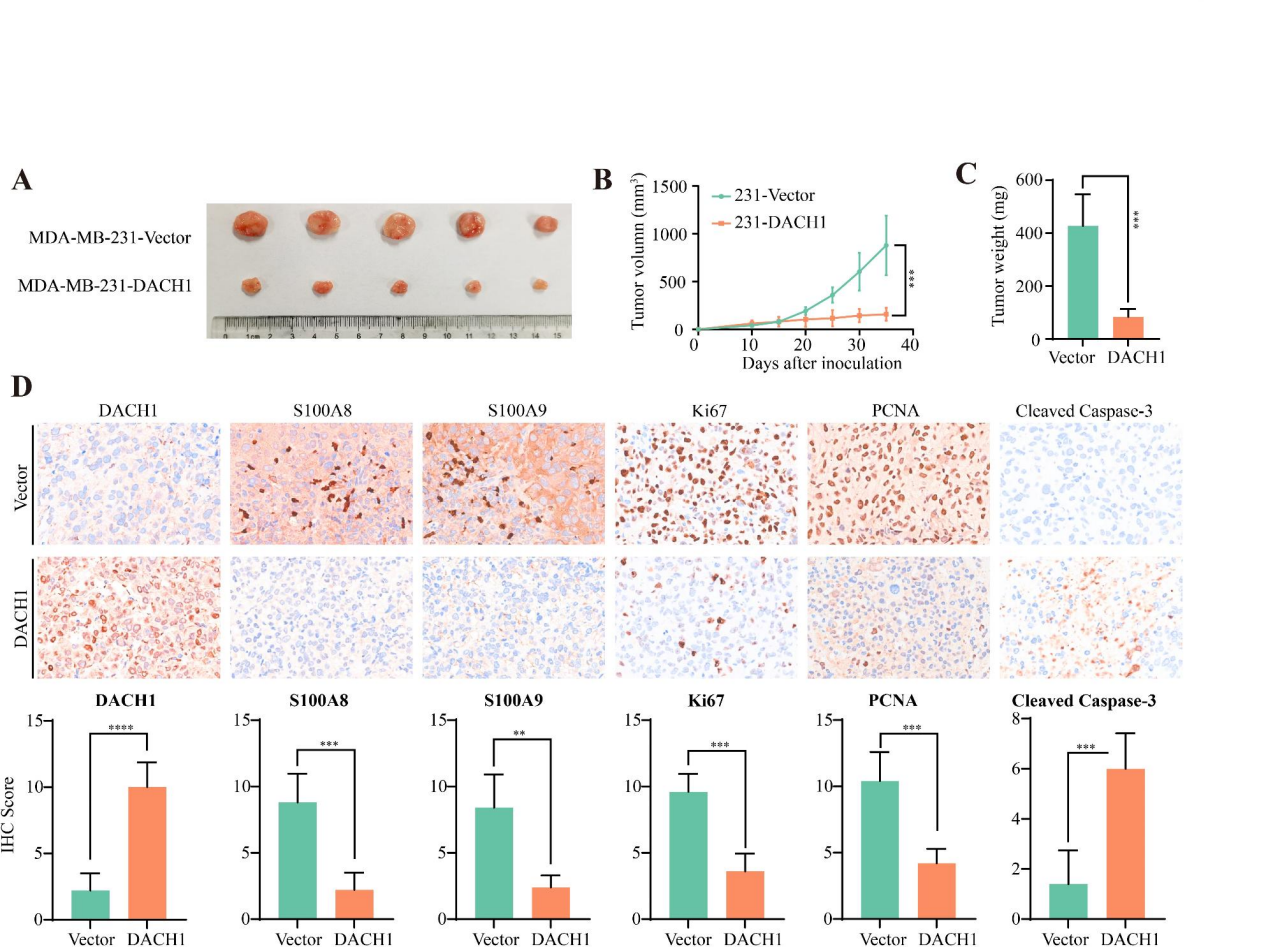
The xenograft tumor assays validating the effects of DACH1 on tumor growth and S100A8/A9 expression. (**A**) The images of MDA-MB-231-vector and MDA-MB-231-DACH1 xenograft tumors (Five mice per group). (**B**) The growth curves of MDA-MB-231-vector and MDA-MB-231-DACH1 xenograft tumors. (**C**) The weights of MDA-MB-231-vector and MDA-MB-231-DACH1 xenograft tumors. (**D**) Immunohistochemical staining to evaluate the effects of DACH1 expression on S100A8/A9, PCNA, Ki67, and cleaved-Caspase 3 levels. The presentative images of anti-DACH1, anti-S100A8, anti-S100A9, anti-Ki67, anti-PCNA, and anti-cleaved-Caspase 3 staining assays

In the xenograft model, BALB/c Nude mice were inoculated with MDA-MB-231-vector or MDA-MB-231-DACH1 cells in the right mammary fat pad. The results showed that sustained DACH1 expression significantly retarded tumor growth and decreased tumor weight (Fig. 11A–C). Besides, we evaluated the expression levels of DACH1, S100A8, S100A9, PCNA, Ki67, and cleaved-caspase 3 in xenograft tumors. Semi-quantitative IHC scores indicated that the protein abundance of S100A8, S100A9, PCNA, and Ki67 were decreased in MDA-MB-231-DACH1 tumors (Fig. 11D). In the meanwhile, the expression level of cleaved-caspase 3 was increased (Fig. 11D). Our results suggested that DACH1 could counteract the expression of S100A8/A9, downregulate proliferation-associated markers (PCNA and Ki67), and upregulate the level of apoptosis-associated markers (cleaved-caspase 3).

## Discussion

S100A8/A9, initially discovered in neutrophils and monocytes, serves as an alarmin during innate immunity activation [17]. It has now become a prominent biomarker for numerous inflammatory conditions and a focal point for drug development in the realm of autoimmune diseases [57, 58]. Furthermore, the levels of S100A8/A9 are markedly elevated in various cancer types, including non-small cell lung cancer [59], gastric cancer [60], colorectal cancer [61], pancreatic cancer [62], and prostate cancer [63]. Frequent genomic rearrangements within the chromosomal region 1q21, where the S100A8 and S100A9 genes cluster, are often detected in human epithelial cancers [64]. Although the precise roles of S100A8/A9 in cancer remain a subject of debate, an increasing body of research suggests that S100A8/A9 may promote cancer progression by activating oncogenic signaling pathways and influencing the tumor immune microenvironment.

On one hand, S100A8/A9 enhances cell migration, invasion, and proliferation by promoting epithelial-mesenchymal transition and activating oncogenic AKT, NF-κB, MAPK, and FAK pathways [25, 31, 65–69]. On the other hand, S100A8/A9 exerts significant effects on the tumor microenvironment. For instance, breast cancer cells overexpress CXCL1/2 to attract myeloid cells into tumors, which in turn generate S100A8/A9 to confer chemoresistance properties to cancer cells and support their survival [70]. Additionally, an increased presence of polymorphonuclear myeloid-derived suppressor cells (PMN-MDSC) in gastric cancer hampers the anti-cancer immune response through S100A8/A9. S100A8/A9 leads to T cell exhaustion by inhibiting T cell proliferation, glycolysis, and cytokine production [71]. Notably, elevated serum levels of S100A8/A9 can serve as a biomarker predicting a poor response to anti-PD-1 antibody treatment [72].

In our study, pooled analysis and immunohistochemical staining indicated that S100A8/A9 was upregulated at both mRNA and protein levels in breast cancer tissues. Moreover, increased S100A8/A9 expression was associated with poor differentiation. Analyses based on public databases and TMAs revealed higher levels of S100A8/A9 in ER-negative, PR-negative, Her2-positive, basal-like, and Her2-overexpressed subtypes. Survival analysis demonstrated that elevated S100A8/A9 predicted a poorer prognosis for breast cancer patients, and the mean expression of S100A8 and S100A9 independently served as a prognostic factor for overall survival in breast cancer patients. Furthermore, serum S100A8 levels were elevated in breast cancer patients compared to those with benign breast nodules. The serum S100A8 level was notably higher in Grade 3, ER-negative, PR-negative, Her2-positive, basal-like, and Her2-overexpressed breast cancer patients. These findings align with previous research, underscoring S100A8/A9 as a risk factor for breast cancer, valuable for prognosis prediction. Particularly, serum S100A8/S100A9 may serve as a suitable surrogate for the detection of tissue S100A8/A9.

Additionally, our study highlights the negative influence of DACH1 on S100A8/A9 expression. Correlation analyses revealed a negative correlation between S100A8/A9 and DACH1 at both the mRNA and protein levels. In vitro experiments indicated that DACH1 overexpression reduced the production of S100A8 in breast cancer cells. Our previous studies have established that DACH1 functions as a transcriptional corepressor by directly inhibiting cytokine transcription through interactions with their promoters [73]. In our present investigation, we found that DACH1 was negatively correlated with S100A8/A9 expression and suppressed the secretion of S100A8. It is plausible that DACH1 may interfere with S100A8/A9 expression at the transcriptional level in a similar manner. It is noteworthy that the combined assessment of S100A8/A9 and DACH1 provides a more precise means of predicting outcomes for breast cancer patients, potentially serving as a promising biomarker for risk classification and prognosis prediction.

## Conclusion

Collectively, S100A8/A9 is not only increased in breast cancers, but also further upregulated in poor differentiation, ER-, PR-, and Her2 + subtypes. Elevated S100A8/A9 predicts poor prognosis of breast cancer patients. High S100A8 in serum is also associated with poor differentiation, loss of hormone receptors, and Her2 + status. In the meanwhile, S100A8/A9 is negatively correlated and regulated by tumor suppressor DACH1. The combination of S100A8/A9 and DACH1 could more effectively differentiate breast cancer patients with poor outcomes.

### Electronic supplementary material

Below is the link to the electronic supplementary material.

**Additional File 1:** Supplementary Tables. **Table S1**. Characteristics of studies involved in meta-analysis. **Table S2**. Correlation between S100A9 expression and clinicopathological features of breast cancer patients in HBreD145Su01. **Table S3**. The clinicopathological features of breast cancer patients used for serum S100A8 detection by ELISA assays.

**Additional File 2: Figure S1**. TCGA data showing relationships between S100A8/A9 mRNA and molecular biomarkers of breast cancers. (A-B) Histogram showing S100A8/A9 mRNA level in ER+, ER-, PR+, PR-, Her2+, and Her2- breast cancers. (C-D) Histogram showing S100A8/A9 mRNA level in different subtypes of breast cancers.

**Additional File 3: Figure S2**. The predictive values of S100A8/A9 mRNA level for the outcomes of breast cancer patients. (A-B) Pooled analysis using GEO datasets showing the relationships between S100A8/A9 mRNA levels and DMFS. (C-D) Pooled analysis using GEO datasets showing the relationships between S100A8/A9 mRNA level and DFS. DMFS: distant metastasis-free survival. DFS: disease-free survival.

## Data Availability

All data and materials generated for this study are included in the article and supplementary files.

## Abbreviations

CCLE: Cancer Cell Line Encyclopedia
CI: confidence interval
DMFS: distant metastasis-free survival
ELISA: enzyme-linked immunosorbent assay
ER: estrogen receptor
Her2: human epidermal growth factor 2
HR: hazard ratio
IHC: Immunohistochemical
MDSC: myeloid-derived suppressor cell
OD: optical densities
OR: odds ratio
OS: overall survival
PMN-MDSC: polymorphonucler myeloid-derived suppressor cell
RFS: relapse-free survival
PR: progesterone receptor
TCGA: The Cancer Genome Atlas
