# Supplementary material for: S100A8/A9 as a risk factor for breast cancer negatively regulated by DACH1
Source: Biomark Res. 2023 Dec 13;11:106. doi: 10.1186/s40364-023-00548-8 (PMC10720252; doi:10.1186/s40364-023-00548-8)
Supplement: Supplementary file 1 — Additional File 1: Supplementary Tables. Table S1. Characteristics of studies involved in meta-analysis. Table S2. Correlation between S100A9 expression and clinicopathological features of breast cancer patients in HBreD145Su01. Table S3. The clinicopathological features of breast cancer patients used for serum S100A8 detection by ELISA assays. [file 40364_2023_548_MOESM1_ESM.docx]

**Table1: characteristic of studies involved in meta-analysis**

| **Reference** | **Year** | **Duration(mo.)** | **Grade** | **Patient number** | **Detection** | **Platform** |
| --- | --- | --- | --- | --- | --- | --- |
| Pawitan Y[1] | 2005 | 102 | 1-3 | 159 | Microarray | Affymetrix Human Genome U133A Array |
| Minn AJ[2] | 2005 | 129 | NA | 99 | Microarray | Affymetrix Human Genome U133A Array |
| Sotiriou C[3] | 2006 | 210 | 1-3 | 189 | Microarray | Affymetrix Human Genome U133A Array |
| Hu Z[4] | 2009 | 96 | 1-3 | 75 | Microarray | Agilent-012097 Human 1A Microarray (V2) G4110B |
| Richardson AL[5] | 2006 | NA | NA | 40 | Microarray | Affymetrix Human Genome U133 Plus 2.0 Array |
| Ivshina AV[6] | 2006 | 153 | 1-3 | 289 | Microarray | Affymetrix Human Genome U133A Array |
| Minn AJ[7] | 2007 | 156 | NA | 58 | Microarray | Affymetrix Human Genome U133A Array |
| Yu K[8] | 2008 | NA | NA | 270 | Microarray | Affymetrix Human Genome U133A Array |
| Lu X[9] | 2008 | NA | 1-3 | 129 | Microarray | Affymetrix Human Genome U133 Plus 2.0 Array |
| Turashvili G[10] | 2007 | NA | NA | 10 | Microarray | Affymetrix Human Genome U133 Plus 2.0 Array |
| Loi S[11] | 2007 | 177 | 1-3 | 327 | Microarray | Affymetrix Human Genome U133A Array |
| Desmedt C[12] | 2007 | 163 | 1-3 | 198 | Microarray | Affymetrix Human Genome U133A Array |
| Loi S[13] | 2008 | 137 | 1-3 | 77 | Microarray | Affymetrix Human Genome U133 Plus 2.0 Array |
| Hennessy BT[14] | 2009 | 106 | 1-3 | 89 | Microarray | Agilent-012097 Human 1A Microarray (V2) G4110B |
| Schmidt M[15] | 2008 | 240 | 1-3 | 200 | Microarray | Affymetrix Human Genome U133A Array |
| Ma XJ[16] | 2009 | NA | 1-3 | 28 | Microarray | Affymetrix Human X3P Array |
| Desmedt C[17] | 2011 | 182 | 1-3 | 120 | Microarray | Affymetrix Human Genome U133 Plus 2.0 Array |
| Symmans WF[18] | 2010 | 196 | NA | 298 | Microarray | Affymetrix Human Genome U133A Array |
| Sircoulomb F[19] | 2010 | 112 | 1-3 | 51 | Microarray | Affymetrix Human Genome U133 Plus 2.0 Array |
| Tabchy A[20] | 2010 | NA | 1-3 | 178 | Microarray | Affymetrix Human Genome U133A Array |
| Kao KJ[21] | 2011 | 169 | NA | 327 | Microarray | Affymetrix Human Genome U133 Plus 2.0 Array |
| Dedeurwaerder S[22] | 2011 | 109 | 1-3 | 88 | Microarray | Affymetrix Human Genome U133 Plus 2.0 Array |
| Sabatier R[23] | 2011 | 222 | 1-3 | 266 | Microarray | Affymetrix Human Genome U133 Plus 2.0 Array |
| Muranen TA[24] | 2011 | 120 | NA | 183 | Microarray | Illumina HumanHT-12 V3.0 expression beadchip |
| Hatzis C[25] | 2011 | 89 | 1-3 | 508 | Microarray | Affymetrix Human Genome U133A Array |
| Filipits M[26] | 2011 | 211 | NA | 277 | Microarray | Affymetrix Human Genome U133A Array |
| Terunuma A[27] | 2014 | 148 | 1-3 | 61 | Microarray | Affymetrix Human Gene 1.0 ST Array |
| Nagalla S[28] | 2013 | 127 | 1-3 | 139 | Microarray | Affymetrix Human Genome U133A Array |
| Tofigh A[29] | 2014 | 145 | 1-3 | 321 | Microarray | Affymetrix Human Gene 1.0 ST Array |

**NA**, not available;

1. Pawitan Y, Bjohle J, Amler L, Borg AL, Egyhazi S, Hall P*, et al.* Gene expression profiling spares early breast cancer patients from adjuvant therapy: derived and validated in two population-based cohorts. Breast Cancer Res. 2005;7:R953-64.

2. Minn AJ, Gupta GP, Siegel PM, Bos PD, Shu W, Giri DD*, et al.* Genes that mediate breast cancer metastasis to lung. Nature. 2005;436:518-24.

3. Sotiriou C, Wirapati P, Loi S, Harris A, Fox S, Smeds J*, et al.* Gene expression profiling in breast cancer: understanding the molecular basis of histologic grade to improve prognosis. J Natl Cancer Inst. 2006;98:262-72.

4. Hu Z, Fan C, Livasy C, He X, Oh DS, Ewend MG*, et al.* A compact VEGF signature associated with distant metastases and poor outcomes. BMC Med. 2009;7:9.

5. Richardson AL, Wang ZC, De Nicolo A, Lu X, Brown M, Miron A*, et al.* X chromosomal abnormalities in basal-like human breast cancer. Cancer Cell. 2006;9:121-32.

6. Ivshina AV, George J, Senko O, Mow B, Putti TC, Smeds J*, et al.* Genetic reclassification of histologic grade delineates new clinical subtypes of breast cancer. Cancer Res. 2006;66:10292-301.

7. Minn AJ, Gupta GP, Padua D, Bos P, Nguyen DX, Nuyten D*, et al.* Lung metastasis genes couple breast tumor size and metastatic spread. Proc Natl Acad Sci U S A. 2007;104:6740-5.

8. Yu K, Ganesan K, Tan LK, Laban M, Wu J, Zhao XD*, et al.* A precisely regulated gene expression cassette potently modulates metastasis and survival in multiple solid cancers. PLoS Genet. 2008;4:e1000129.

9. Lu X, Lu X, Wang ZC, Iglehart JD, Zhang X, Richardson AL. Predicting features of breast cancer with gene expression patterns. Breast Cancer Res Treat. 2008;108:191-201.

10. Turashvili G, Bouchal J, Baumforth K, Wei W, Dziechciarkova M, Ehrmann J*, et al.* Novel markers for differentiation of lobular and ductal invasive breast carcinomas by laser microdissection and microarray analysis. BMC Cancer. 2007;7:55.

11. Loi S, Haibe-Kains B, Desmedt C, Lallemand F, Tutt AM, Gillet C*, et al.* Definition of clinically distinct molecular subtypes in estrogen receptor-positive breast carcinomas through genomic grade. J Clin Oncol. 2007;25:1239-46.

12. Desmedt C, Piette F, Loi S, Wang Y, Lallemand F, Haibe-Kains B*, et al.* Strong time dependence of the 76-gene prognostic signature for node-negative breast cancer patients in the TRANSBIG multicenter independent validation series. Clin Cancer Res. 2007;13:3207-14.

13. Loi S, Haibe-Kains B, Desmedt C, Wirapati P, Lallemand F, Tutt AM*, et al.* Predicting prognosis using molecular profiling in estrogen receptor-positive breast cancer treated with tamoxifen. BMC Genomics. 2008;9:239.

14. Hennessy BT, Gonzalez-Angulo AM, Stemke-Hale K, Gilcrease MZ, Krishnamurthy S, Lee JS*, et al.* Characterization of a naturally occurring breast cancer subset enriched in epithelial-to-mesenchymal transition and stem cell characteristics. Cancer Res. 2009;69:4116-24.

15. Schmidt M, Bohm D, von Torne C, Steiner E, Puhl A, Pilch H*, et al.* The humoral immune system has a key prognostic impact in node-negative breast cancer. Cancer Res. 2008;68:5405-13.

16. Ma XJ, Dahiya S, Richardson E, Erlander M, Sgroi DC. Gene expression profiling of the tumor microenvironment during breast cancer progression. Breast Cancer Res. 2009;11:R7.

17. Desmedt C, Di Leo A, de Azambuja E, Larsimont D, Haibe-Kains B, Selleslags J*, et al.* Multifactorial approach to predicting resistance to anthracyclines. J Clin Oncol. 2011;29:1578-86.

18. Symmans WF, Hatzis C, Sotiriou C, Andre F, Peintinger F, Regitnig P*, et al.* Genomic index of sensitivity to endocrine therapy for breast cancer. J Clin Oncol. 2010;28:4111-9.

19. Sircoulomb F, Bekhouche I, Finetti P, Adelaide J, Ben Hamida A, Bonansea J*, et al.* Genome profiling of ERBB2-amplified breast cancers. BMC Cancer. 2010;10:539.

20. Tabchy A, Valero V, Vidaurre T, Lluch A, Gomez H, Martin M*, et al.* Evaluation of a 30-gene paclitaxel, fluorouracil, doxorubicin, and cyclophosphamide chemotherapy response predictor in a multicenter randomized trial in breast cancer. Clin Cancer Res. 2010;16:5351-61.

21. Kao KJ, Chang KM, Hsu HC, Huang AT. Correlation of microarray-based breast cancer molecular subtypes and clinical outcomes: implications for treatment optimization. BMC Cancer. 2011;11:143.

22. Dedeurwaerder S, Desmedt C, Calonne E, Singhal SK, Haibe-Kains B, Defrance M*, et al.* DNA methylation profiling reveals a predominant immune component in breast cancers. EMBO Mol Med. 2011;3:726-41.

23. Sabatier R, Finetti P, Cervera N, Lambaudie E, Esterni B, Mamessier E*, et al.* A gene expression signature identifies two prognostic subgroups of basal breast cancer. Breast Cancer Res Treat. 2011;126:407-20.

24. Muranen TA, Greco D, Fagerholm R, Kilpivaara O, Kampjarvi K, Aittomaki K*, et al.* Breast tumors from CHEK2 1100delC-mutation carriers: genomic landscape and clinical implications. Breast Cancer Res. 2011;13:R90.

25. Hatzis C, Pusztai L, Valero V, Booser DJ, Esserman L, Lluch A*, et al.* A genomic predictor of response and survival following taxane-anthracycline chemotherapy for invasive breast cancer. Jama. 2011;305:1873-81.

26. Filipits M, Rudas M, Jakesz R, Dubsky P, Fitzal F, Singer CF*, et al.* A new molecular predictor of distant recurrence in ER-positive, HER2-negative breast cancer adds independent information to conventional clinical risk factors. Clin Cancer Res. 2011;17:6012-20.

27. Terunuma A, Putluri N, Mishra P, Mathe EA, Dorsey TH, Yi M*, et al.* MYC-driven accumulation of 2-hydroxyglutarate is associated with breast cancer prognosis. J Clin Invest. 2014;124:398-412.

28. Nagalla S, Chou JW, Willingham MC, Ruiz J, Vaughn JP, Dubey P*, et al.* Interactions between immunity, proliferation and molecular subtype in breast cancer prognosis. Genome Biol. 2013;14:R34.

29. Tofigh A, Suderman M, Paquet ER, Livingstone J, Bertos N, Saleh SM*, et al.* The prognostic ease and difficulty of invasive breast carcinoma. Cell Rep. 2014;9:129-42.

**Table S2** Correlation between S100A9 expression and clinicopathological features of breast cancer patients in HBreD145Su01.

| **Variables** | **n** | **S100A9 (High)** | **S100A9 (Low)** | ***P* value** |
| --- | --- | --- | --- | --- |
| **Tumor size** |  |  |  | 0.489 |
| T3 | 10 | 8 | 2 |  |
| T1-T2 | 97 | 62 | 35 |  |
| **Lymph node** |  |  |  | 0.678 |
| N+ | 75 | 47 | 28 |  |
| N- | 38 | 26 | 12 |  |
| **Stage** |  |  |  | 0.219 |
| Ⅲ | 41 | 30 | 11 |  |
| Ⅰ-Ⅱ | 72 | 43 | 29 |  |
| **ER** |  |  |  | <0.0001 |
| ER+ | 72 | 37 | 35 |  |
| ER- | 34 | 31 | 3 |  |
| **PR** |  |  |  | <0.0001 |
| PR+ | 63 | 29 | 34 |  |
| PR- | 43 | 39 | 4 |  |
| **Her2(FISH)** |  |  |  | 0.048 |
| Her2+ | 33 | 26 | 7 |  |
| Her2- | 73 | 42 | 31 |  |

**Table S3** The clinicopathological features of breast cancer patients used for serum S100A8 detection by ELISA assays.

| **Variables** | **n** |
| --- | --- |
| **Tumor location** |  |
| Right | 37 |
| Left | 28 |
| **Grade** |  |
| 1-2 | 47 |
| 3 | 18 |
| **ER** |  |
| ER+ | 46 |
| ER- | 19 |
| **PR** |  |
| PR+ | 44 |
| PR- | 21 |
| **Her2** |  |
| Her2+ | 25 |
| Her2- | 40 |
| **Subtype** |  |
| Luminal | 34 |
| Her2-overexpression | 25 |
| Basal | 6 |
