# Supplementary figures and images for: S100A8/A9 as a risk factor for breast cancer negatively regulated by DACH1
Source: Biomark Res. 2023 Dec 13;11:106. doi: 10.1186/s40364-023-00548-8 (PMC10720252; doi:10.1186/s40364-023-00548-8)

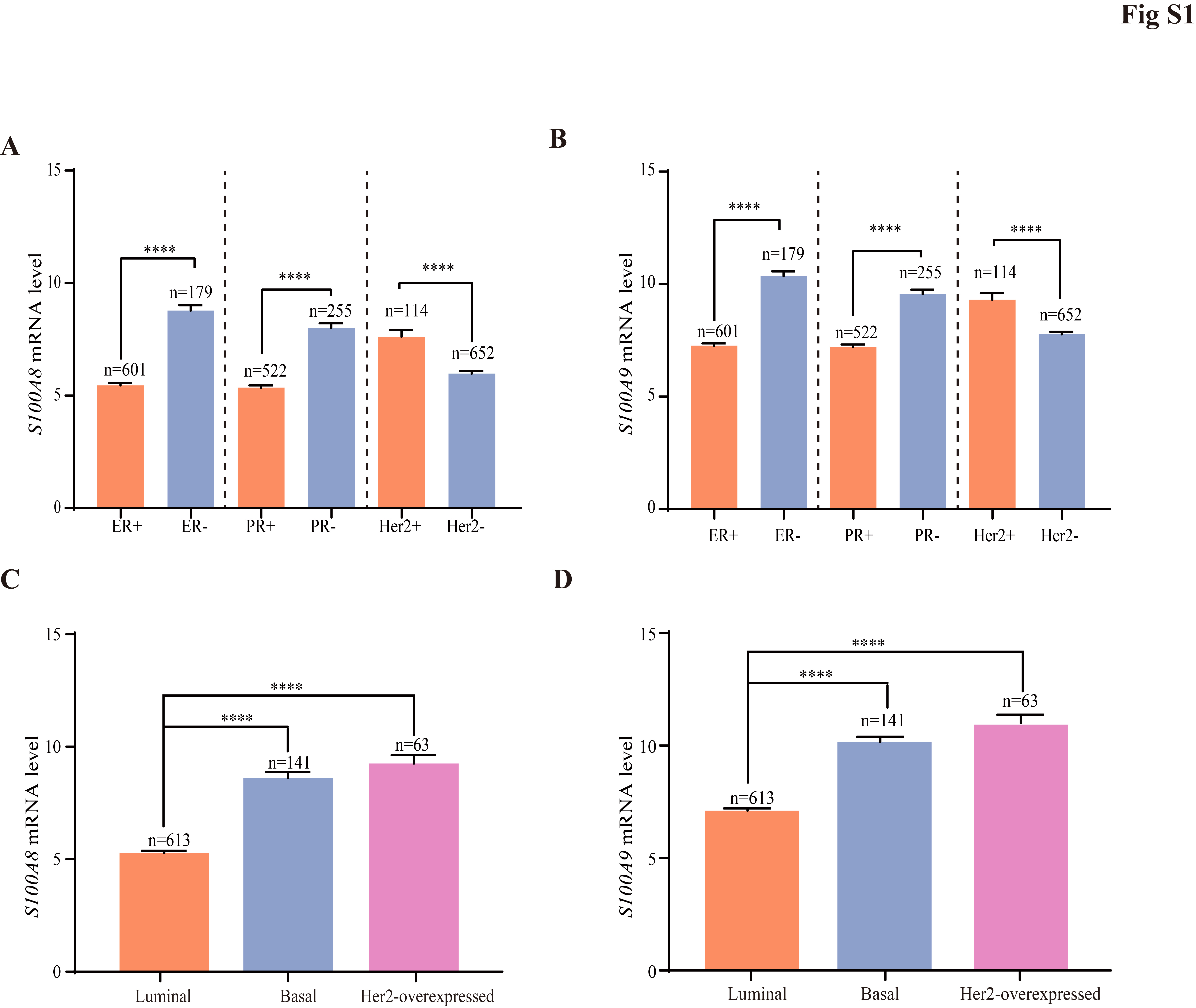

Supplement: Supplementary file 2 — Additional File 2: Figure S1. TCGA data showing relationships between S100A8/A9 mRNA and molecular biomarkers of breast cancers. (A-B) Histogram showing S100A8/A9 mRNA level in ER+, ER-, PR+, PR-, Her2+, and Her2- breast cancers. (C-D) Histogram showing S100A8/A9 mRNA level in different subtypes of breast cancers. [file 40364_2023_548_MOESM2_ESM.jpg]

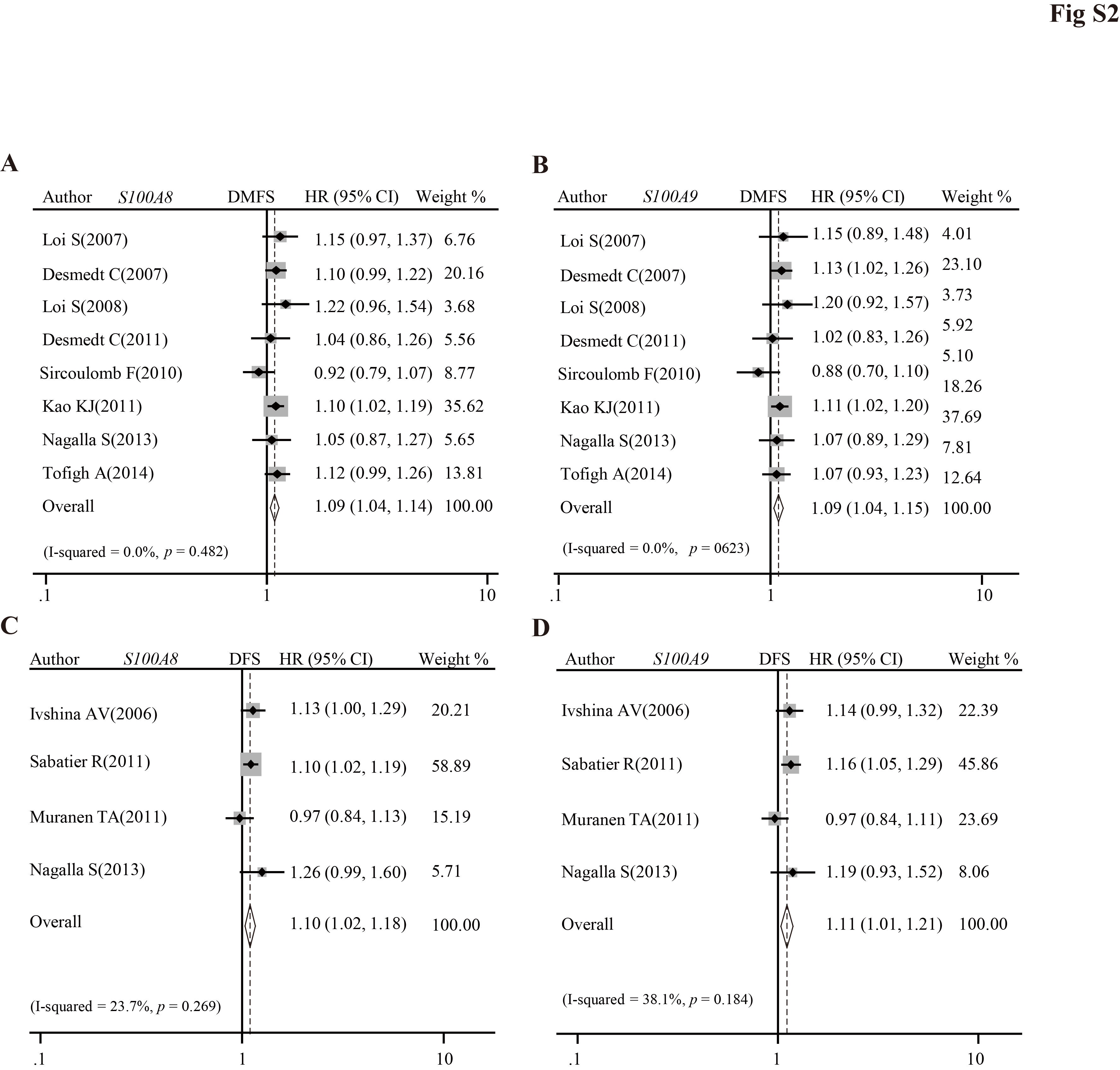

Supplement: Supplementary file 3 — Additional File 3: Figure S2. The predictive values of S100A8/A9 mRNA level for the outcomes of breast cancer patients. (A-B) Pooled analysis using GEO datasets showing the relationships between S100A8/A9 mRNA levels and DMFS. (C-D) Pooled analysis using GEO datasets showing the relationships between S100A8/A9 mRNA level and DFS. DMFS: distant metastasis-free survival. DFS: disease-free survival. [file 40364_2023_548_MOESM3_ESM.jpg]
